# A Randomized Double-Blind, Cross-Over Trial of very Low-Calorie Diet in Overweight Migraine Patients: A Possible Role for Ketones?

**DOI:** 10.3390/nu11081742

**Published:** 2019-07-28

**Authors:** Cherubino Di Lorenzo, Alessandro Pinto, Roberta Ienca, Gianluca Coppola, Giulio Sirianni, Giorgio Di Lorenzo, Vincenzo Parisi, Mariano Serrao, Alessandra Spagnoli, Annarita Vestri, Jean Schoenen, Lorenzo M Donini, Francesco Pierelli

**Affiliations:** 1 IRCCS–Fondazione Don Carlo Gnocchi, 20121 Milan, Italy; 2 Department of Experimental Medicine, Sapienza University of Rome, 00161 Roma, Italy; 3 Department of Medico-Surgical Sciences and Biotechnologies, Sapienza University of Rome Polo Pontino, 04100 Latina, Italy; 4 Associazione Eupraxia, Dietary Section, 00171 Rome, Italy; 5 Laboratory of Psychophysiology and Cognitive Neuroscience, Department of Systems Medicine, University of Rome Tor Vergata, 00133 Rome, Italy; 6 IRCCS Fondazione Santa Lucia, 00142 Rome, Italy; 7 IRCCS–Fondazione Bietti, 00198 Rome, Italy; 8 Department of Public Health and Infectious Diseases, Sapienza University of Rome, 00161 Rome, Italy; 9 Headache Research Unit, University Department of Neurology CHR, Citadelle Hospital, University of Liège, 4000 Liège, Belgium; 10 IRCCS–Neuromed, 86077 Pozzilli (IS), Italy

**Keywords:** ketosis, attacks frequency, migraine, ketone bodies, weight loss, low-carbohydrate, low-calorie

## Abstract

Here we aimed at determining the therapeutic effect of a very low-calorie diet in overweight episodic migraine patients during a weight-loss intervention in which subjects alternated randomly between a very low-calorie ketogenic diet (VLCKD) and a very low-calorie non-ketogenic diet (VLCnKD) each for one month. In a nutritional program, 35 overweight obese migraine sufferers were allocated blindly to 1-month successive VLCKD or VLCnKD in random order (VLCKD-VLCnKD or VLCnKD-VLCD). The primary outcome measure was the reduction of migraine days each month compared to a 1-month pre-diet baseline. Secondary outcome measures were 50% responder rate for migraine days, reduction of monthly migraine attacks, abortive drug intake and body mass index (BMI) change. Only data from the intention-to-treat cohort (*n* = 35) will be presented. Patients who dropped out (*n* = 6) were considered as treatment failures. Regarding the primary outcome, during the VLCKD patients experienced −3.73 (95% CI: −5.31, −2.15) migraine days respect to VLCnKD (*p* < 0.0001). The 50% responder rate for migraine days was 74.28% (26/35 patients) during the VLCKD period, but only 8.57% (3/35 patients) during VLCnKD. Migraine attacks decreased by −3.02 (95% CI: −4.15, −1.88) during VLCKD respect to VLCnKD (*p* < 0.00001). There were no differences in the change of acute anti-migraine drug consumption (*p* = 0.112) and BMI (*p* = 0.354) between the 2 diets. A VLCKD has a preventive effect in overweight episodic migraine patients that appears within 1 month, suggesting that ketogenesis may be a useful therapeutic strategy for migraines.

## 1. Introduction

The ketogenic diet (KD) constitutes high-fat, adequate protein, and low-carbohydrate, and has been proven to be efficacious for the treatment of drug-resistant epilepsy [1]. Recently, KD showed promising results for treating other neurological conditions (for example, glioblastomamultiforme, malignant glioma, and Alzheimer’s disease) [2]. Migraine is one of the many pathological conditions that has been reported to benefit from KD. The initial reports of the beneficial effect of KD on migraine date back to the 1920s. In 1928, Schnabel reported his experience with KD in patients with migraine [3]. In 1930, Barborka, one of the pioneers who popularized KD among adults, reported outstanding improvement of migraines following the initiation of KD in a study of 50 patients [4]. In 2006, a regimen of low-carbohydrate and low-fat diets based on food replacements for rapid weight loss in a cohort of patients with chronic migraine demonstrated concomitant improvement of their migraines. The author identified in diet-induced ketosis the reason of the improvement [5]. Similarly, we demonstrated an analogous migraine-alleviating effect on a couple of overweight sisters who experienced migraines, and who followed a cyclic low-fat low-carbohydrate diet. The diet was followed in 3 repetitive phases, one phase every 3 months, during which the patients exhibited stable ketosis, as confirmed by urinary strips for the detection of ketosis, and reported improvements of their migraines [6].

The aforementioned diets are not the conventional high-fat diets similar to those classically adopted to treat neurological conditions [7], since these diets use “quasi-fasting induced ketosis”. Physiologically, fasting also induces metabolic ketosis [8] in a faster way, and leads to a higher ketone concentration than low carbohydrate high fat diet. When an overweight individual severely restricts his or her caloric intake, the body induces itself to a state of fasting and exhibits mild ketosis [8]. These diets propagated for drastic slimming are called very low-calorie diet (VLCD). Although these diets can only be followed for limited periods because of their extreme caloric restriction, they can induce a high rate of weight-loss with long-term maintenance [9], especially if practiced along with an active follow-up treatment [10]. Furthermore, the protein sparing modified fast (PSMF), a modified variant of VLCD, was designed to exploit quasi-fasting induced ketosis to preserve lean body mass when following major weight loss programs, as widely studied and described by Dr. George Blackburn [11]. Because of their ability to induce ketosis by the mobilization of fatty acids from adipose tissue (physiological states of prolonged fasting), PSMF protocols are also called very low-calorie ketogenic diets (VLCKDs). Based on the studies by Blackburn, the concept of VLCKD was popularized to achieve weight loss and manage metabolic disorders [12,13,14].

Following the case reports by Strahlman and Di Lorenzo et al. [5,6], we compared overweight individuals experiencing migraines and who followed VLCKD to patients prescribed with a standard non-ketogenic hypocaloric diet in a proof-of-concept, prospective, observational pilot study, and found a beneficial effect of VLCKD on various clinical features of migraine features [15]. Because of the lack of randomization and blinding, the encouraging results described herein needed to be further validated by a randomized, controlled, double-blind trial. 

Considering the negative impact of obesity on migraine disability [16], we conducted our trial in overweight individuals experiencing episodic migraines who participated in a nutritional low-calorie program. Using a double-blind cross-over design, we analyzed the effects of a VLCKD by comparing it with the effect of a very low-calorie non-ketogenic diet (VLCnKD) which was followed by each patient for 1 month randomly. This trial protocol was intended to allow the resolution of the effect on migraine of caloric restriction and metabolic ketosis. 

## 2. Materials and Methods 

The trial protocol, written according to the European Medicines Agency Good Clinical Practice and consistent with the principles set out in the Declaration of Helsinki, was approved by the Policlinico “Umberto I” ethics committee. Patients provided written informed consent. The study has been registered with clinicaltrials.gov (https://clinicaltrials.gov/show/NCT03076060) ClinicalTrials.gov identifier: NCT03076060.

This was a single-center, randomized, double-blind, controlled, cross-over, phase 2 trial of a nutritional intervention conducted between January and October 2017. The Department of Medico-Surgical Sciences and Biotechnologies provided meal replacements for the patients and neurological supervision of the study. The Department of Experimental Medicine, Physiopathology, Food Science, and Endocrinology section organized the nutritional management of patients, and the Department of Public Health performed the data analyses. All departments belong to the Sapienza University of Rome.

At the end of a 4-week baseline period of free normal diet, eligible patients received a VLCD and were assigned in a 1:1 ratio to two successive 4-week periods of a VLCKD or VLCnKD in a randomized order. The VLCD is an extreme (≤800 kcal), time-restricted, nutritional protocol that mimics the effects of fasting without the side effects of starvation, to treat severe obesity [17]. Given its carbohydrate content, it could, or not, induce metabolic ketosis [11]. Both diets were based on food replacements delivered to the patients’ home by the product supplier (Kalibra^®^ by S.D.M. srl, Savigliano (CN), Italy). The composition of both diets was as follows: 

-VLCKD: in agreement with the European Food Safety Authority (EFSA) [18], this diet contained a minimum protein content based on a Population Reference Intake for protein adjusted for the overweight (≥75 g/day), a very limited carbohydrate content (30–50 g/day), a fixed amount of fats (20 g/day mainly from olive oil), and micronutrients to fulfill the Dietary Reference Intake (DRI) (Table 1).

-VLCnKD: protein ≅ 50 g/day, a carbohydrate intake ≥70 g/day, a fixed amount of fats (20 g/day mainly from olive oil), and micronutrients.

**Figure 1:**
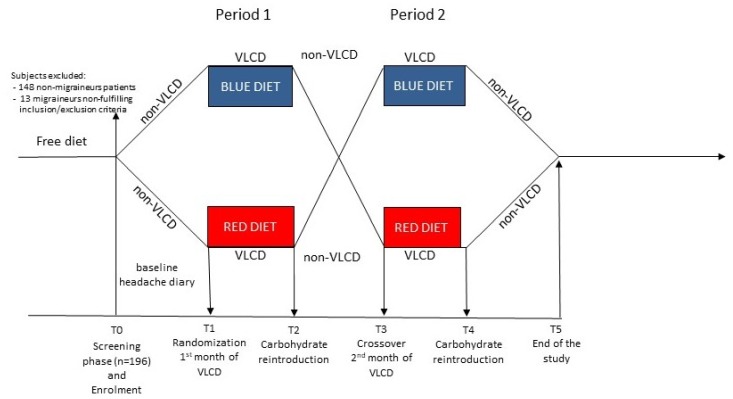
Non-VLCD = normoglycaemic diet; VLCD = very low-calorie diet, supplemented by nutraceutical integrators (RED DIET and BLUE DIET are the 2 different treatments, one ketogenic, the other non-ketogenic).

The trial had five phases (Figure 1): (1) a 4-week screening baseline with non-VLCD (T0); (2) after randomization to the double-blind part of the trial, a 1st 4-week nutritional intervention phase (one of the two VLCD), with a control visit at the end of the 2nd week (T1); (3) a 4-week phase of progressive carbohydrate reintroduction and caloric increase in a non-VLCD (T2); (4) the cross-over to the 2nd 4-week nutritional intervention phase (the other VLCD), with a control visit at the end of the second week (T3); (5) the 2nd 4-week progressive carbohydrate reintroduction and caloric increase in a non-VLCD (T4).

The 4-weeks non-VLCDs followed by patients after the VLCD months were characterized by a week-by-week caloric and carbohydrate progressive reintroduction from breakfast to dinner, to wean themselves from the state of ketosis and/or caloric restriction. Macronutrients and calories were personalized to patients’ needs.

### 2.1. Randomization and Blinding

Randomization was based on a predefined randomization schedule generated by using the Web site randomization.com (http://www.randomization.com). The two different VLCDs were identified by a color code (blue and red) assigned by a delegate of the supplier who was blinded to the patients’ clinical characteristics. The patients and investigators were able to see the color label of the diets, but they were not aware of the color code for each diet. 

### 2.2. Study Population

One-hundred and ninety-six patients attending the Food Science and Endocrinology Clinic at the Policlinico Umberto I of Rome to receive a nutritional intervention for overweight or obesity, were screened with ID Migraine [19]. Migraine sufferers, once their diagnosis was confirmed by a headache neurologist (C.D.L., G.C.), were thereafter enrolled in the trial. Apart from migraine, obesity and prediabetes, participants were generally healthy individuals. Participants eligible for trial participation were overweight/obese adults (Body Mass Index (BMI) > 25), aged 18 to 65 years, who had at least 12 months’ history of migraines with or without aura, defined according to the criteria of the International Classification of Headache Disorders, 3rd edition beta version (ICHD-3b) [20]. Patients had to have a history of between 2 and 14 migraine days per month on average during the 3-months preceding the trial and during the 4-weeks baseline phase. Exclusion criteria were: migraine onset after 50 years of age, co-morbidity with other primary (tension-type headache, trigeminal autonomic cephalalgias, other primary headache disorders) or secondary headaches (e.g., headaches attributed to trauma or injury, vascular or non-vascular intracranial disorders, substances or its withdrawal, infections, alterations of homeostasis), hemiplegic migraine, use of preventive migraine -medication or neurostimulation devices in the last 3 months before the screening visit, overuse of acute anti-migraine drugs (as defined in ICHD-3b), known psychiatric comorbidities (assessed by anamnesis), pregnancy or lactation, type I diabetes, use of corticosteroids, use of non- K^+^ sparing diuretics, kidney and liver failure.

### 2.3. Outcome Measures

Vital signs (stable sitting blood pressure and pulse frequency), height and weight, waist circumference, and skinfold calipers were determined at the beginning and at the end of each diet period. Early morning fasting blood tests were performed at T1, T2 and T4 (data were reported as Supplementary Materials Table S1 and Figure S1). We did not ask patients to check for ketone bodies in urine because of the double-blind nature of the study (but laboratory detected them as standard part of their complete urinary test protocol).

During the whole study, patients completed a headache diary with information about headache date, time of onset and resolution, pain severity, associated symptoms, and use of abortive therapies. Safety was monitored throughout the trial by the reporting of adverse events, including evaluation of laboratory values and vital signs.

The primary end point was the change in mean number of migraine days per month during the two blinded phases of VLCDs (VLCKD and VLCnKD). A migraine day was defined as any calendar day on which the patient had onset, continuation, or recurrence of a qualified migraine as recorded in the diary [20]. The first-tier secondary end points were the change in mean number of migraine attacks (defined as a pain period preceded and followed by at least 24 h without pain) per month and the change from the baseline in mean number of doses of acute medication monthly used. The second-tier secondary end point was the 50% responder rate (the proportion of patients having at least a 50% reduction of mean number of migraine days) [21]. The third-tier secondary end point was a change in BMI. 

### 2.4. Statistical Analysis

According to our previous open study data [15], we performed a sample size calculation. A sample of 29 patients was required in this two-treatment crossover study to detect, with a probability of 90%, a treatment difference at a two-sided 0.05 significance level, if the true difference between treatments is 4.860 units. This is based on the assumption that the within-patient standard deviation of the response variable was 5.456. The sample size was increased by 20% (plausible drop-out value); thus, we decided to enroll 35 participants.

Patient characteristics were described using mean (SD) and median with 25th (Q1) and 75th (Q3) percentiles for continuous and frequencies (and percentages) for dichotomous variables. In order to study the behavioral of clinical variables of interest for our purposes (monthly number of migraine days, number of migraine attacks, doses of acute symptomatic medications, BMI) during VLCD phases we calculated changes between T1 and T2 (period 1) and between T3 and T4 (period 2). To analyze primary and continuous secondary end points, a linear mixed model (LMM) was used with VLCD treatment period (period 1 vs. period 2) and treatment (VLCKD vs. VLCnKD) as fixed and participants as random effect, adjusted for age, BMI at baseline, gender [repeated measure is missing]. Data were analyzed, blinded to treatment, using R software. A *p*-value < 0.05 was considered statistically significant.

For the secondary end point of responder rate, we calculated the frequency of responders (patients that have had 50% or greater reduction in mean migraine days per month).

The responder rate was analyzed on an intention-to-treat (ITT) basis. The ITT population included all randomized patients who started a VLCD diet. Missing data were imputed by assuming failure (not responding). The number of patients needed to treat (NNT) was determined with the formula: [(responders to VLCKD/Total patients) − (responders to VLCnKD/Total patients)]^−1^.

## 3. Results

### 3.1. Participants

Out of a total of 196 overweight/obese screened patients, 48 received a diagnosis of migraine with or without aura, and were screened for the exclusion criteria; 35 patients underwent randomization (causes of exclusion were the co-existence of other forms of headache and/or the presence of known psychiatric comorbidities) and 29 patients (82.86%) completed the study. Eighteen of the enrolled patients have neither used migraine-preventive medications, nor have referred to a headache clinic in the past. During the baseline phase, the whole trial group had 29 women (82.86%) and 7 men (17.14%), with a mean age of 43 (± 10) years, with a mean of 7 (± 3) migraine days per month, with a mean of 5 (± 2) migraine attacks per month, with a mean of 5 (± 4) doses of symptomatic drugs per month, and with a mean of BMI of 35.40 (± 6.25). At study baseline, no significant differences in sociodemographic and clinical variables emerged between the two sequence groups: the group that starts with the red diet and then switches to blue diet (R→B) versus the group that starts with the blue diet and then switches to red diet (B→R) (Table 2).

Six patients dropped out in the first month of VLCD diet. All dropout patients were assigned to the sequence B ≥ R. They complained of the excessive hardness of the blue diet, judging it to be impossible to carry on.

Blood glucose values did not differ between the baseline and those observed after the two dietary regimens (4.8 ± 0.5 mmol/L at the baseline; 4.8 ± 0.5 mmol/L after Blue diet (*p* = 1.0); 4.7 ± 0.5 mmol/l after Red diet (*p* = 0.233). On the other hand, insulinemia values decreased significantly after following the red diet, but only a downward trend that did not reach statistical significance was observed after the blue diet (77.44 ± 36.67 pmol/L at the baseline; 58.82 ± 30.97 pmol/L after the blue diet, *p* = 0.12; 54.45 ± 23.96 pmol/L after the red diet, *p* = 0.001). There was no difference between patients following the red and blue diets in terms of blood sugar (*p* = 0.181) and insulinemia (*p* = 0.939). 

When, at the end of the study, the double blind was open, the colors were associated with the kind of diet: the red diet was VLCKD, and the blue one was the VLCnKD.

In order to respect the double-blind nature of the study, laboratory provided data about urinary ketosis only at the end of the study. At the baseline, urinary ketosis was not found in patients; only one patient out of 29 (3.4%) tested positive in a urinary ketosis test at the end of the month of following the blue diet (value 0.5 mmol/L); at the end of the month of the red diet, 22 out of 29 (75.9%) patients were found to be positive at the urinary test for ketosis (range 0.5–4.5 mmol/L).

### 3.2. Efficacy

Detailed results of LMM are reported in Table 3. 

**Figure 2:**
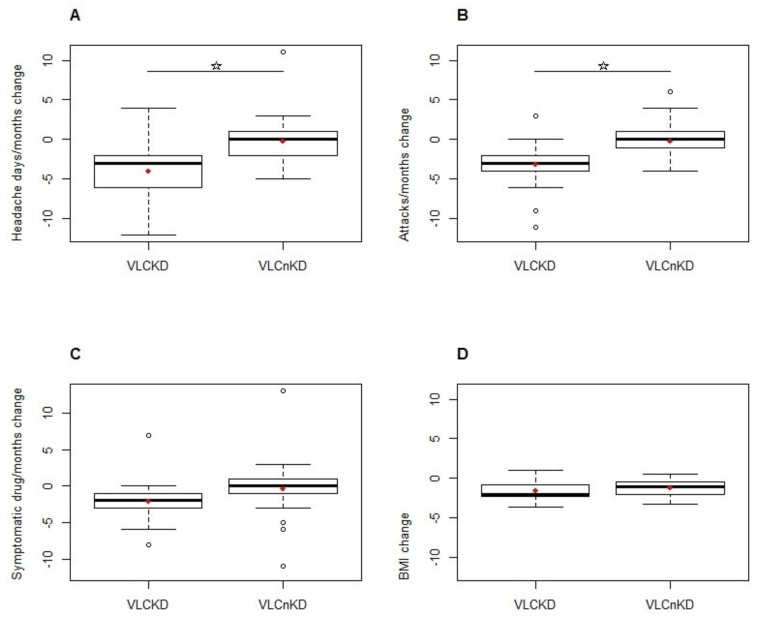
Box-plots depict treatment effects (VLCKD vs. VLCnKD) on changes in Headache days/months (**A**), Attacks/months (**B**), Symptomatic drugs/months (**C**) and BMI (**D**). In the figures: solid lines indicate median values; boxes show 25% to 75% range; whiskers include non-outlier range; circles indicate outlier and extreme values; red diamonds represent mean values. VLCKD: ketogenic very low-calorie diet; VLCnKD: non-ketogenic very low-calorie diet. In figures A and B the horizontal bar and star indicate statistical significance. Star indicates statistical significance. Circles indicate the presence of outliers’ values.

The primary outcome was the change in mean number of monthly migraine days during the two blinded phases of VLCD. By LMM, the only independent variable with a significant effect was the fixed factor “Treatment” (*p* < 0.0001). During the VLCKD patients experienced −3.73 (95% CI: −5.31, −2.15) migraine days respect to VLCnKD treatment (Figure 2A).

The first-tier secondary end points were the change in mean number of monthly migraine attacks and the change in mean number of monthly doses of acute symptomatic medications.

Regarding the change in the mean number of migraine attacks per month, by LMM, the only independent variable with a significant effect was the fixed factor “Treatment” (*p* < 0.00001). During the VLCKD, patients experienced −3.02 (95% CI: −4.15, −1.88) migraine attacks in comparison to VLCnKD treatment (Figure 2B).

Regarding the change in the mean number of doses of acute symptomatic medications per month, by using LMM, no variable entered as significant predictor in the model. The fixed factor “treatment” did not reach the significance threshold (*p* = 0.112), with patients during the VLCKD treatment consuming −1.49 (95% CI: −3.19, 0.22) doses of acute symptomatic drugs compared to VLCnKD treatment (Figure 2C).

The monthly change in BMI was the third-tier secondary end point. By using LMM, the fixed factor “treatment” was not significant (*p* = 0.354) (Figure 2D), while the fixed factor “period” had a significant effect (*p* < 0.00001). During period 2, patients showed a BMI value that was 1.12 (95% CI: 0.726, 1.511) higher than during period 1. 

Regarding the responders’ rate (number of patients with a reduction of at least 50% headache days), 26 out of 35 patients (74.28% of intention to treat (ITT) patients) responded to VLCKD; 3 (8.57% of ITT patients) responded to VLCnKD. 

The NNT comprised 2 participants. Safety and adverse events: mild constipation (12 patients) and muscle cramps (1 patient) were the only adverse events observed during the protocol. The first was treated by hydration and, occasionally, the use of macrogol (aka polyethylene glycol); the second was treated by increasing potassium supplementation during the diet.

## 4. Discussion

The most striking result of our study is that a 4-week period VLCKD, despite inducing similar weight loss and glycemic profile, was significantly more effective than VLCnKD in preventing migraine attacks, as evidenced by a decrease in the frequency of migraine days and attacks, and a greater than 50% response rate. 

It is worth underlining that this result was obtained through a direct comparison of two isocaloric diets, containing the same nutraceuticals and a similar amounts and types of fats. Hence, the only variables that can explain the anti-migraine effect seem to be the restriction in carbohydrates and the ensuing metabolic ketosis. Another noteworthy feature of the trial is that the beneficial therapeutic effect appeared within only 4 weeks, which is a rather early onset of action for preventive migraine treatments. Moreover, the VLCKD is overall well tolerated. In fact, the dropouts in our trial occurred during VLCnKD, in which 6 patients refused to try the other very low calorie diet. We assume that the VLCnKD was not tolerated by these patients because of the severe caloric restriction (similar to the other one), but also because they did not benefit from the protective effect of proteins and ketones on hunger [22] and headaches.

As mentioned, the VLCKD can lead to mild ketosis [8]. After breaking the blind, urinary tests at the end of the month of diet ketones were detected in 75.9% of subjects who underwent VLCKD and in 3.4% of subjects who underwent the VLCnKD, strongly supporting the idea that VLCKD is a KD. 

The mechanisms by which KD improves migraines may be manifold. Weight loss in overweight/obese migraine sufferers was found to reduce attack frequency in some studies [23]. Our results, however, are not explainable by weight loss, because it was similar for both VLCDs, and there was no beneficial effect on migraines with the VLCnKD. Knowing their role in migraine pathophysiology [24,25], decreasing insulin and glucose levels could explain our results. However, compared to the baseline, glycaemia non-significantly decreased after the two diets, while only insulin levels significantly decreased after following the red diet, leaving the possibility that a reduced insulinemia induced by VLCKD could at least partially explain its clinical efficacy, although there were no differences between red and blue.

We have previously shown that KD normalizes the habituation deficit of evoked potentials [26,27] that is typically observed in migraines between attacks, and reflects cerebral hyperresponsivity [28]. Furthermore, in animal experiments, ketones were shown to effectively counteract various phenomena involved in migraine pathophysiology; they reduce propagation of cortical spreading depression (CSD), the likely culprit for the migraine aura [29], dampen neuroinflammation [30], and protect against oxidative stress [29]. Ketone bodies are also able to influence the brain’s mitochondrial energy metabolism that is known to be impaired in migraine [31]. The mismatch between low ATP levels and the excessive neuronal activation during the interictal phase [32,33] could lead to a metabolic disequilibrium which is able to facilitate CSD and to activate the trigeminovascular system, and thus, to trigger a migraine attack [34,35]. Ketone bodies, in particular beta-hydroxybutyrate (BHB) and acetoacetate (AcAc), cross the blood-brain barrier and penetrate into astrocytes and neurons via monocarboxylate transporters. Ketones could improve mitochondrial energy metabolism in migraine sufferers by modulating mitochondrial gene expression [36], in particular mitochondrial 3-hydroxy-3-methylglutaryl-CoA synthase [37], by reducing free radical production and increasing NADH oxidation [38]. BHB, for instance, provides an alternative substrate to glucose for oxidative phosphorylation; it is converted to AcAc, and subsequently, in the mitochondria, to acetyl-CoA, which feeds into the Krebs cycle to produce ATP. To determine which of the ketone bodies are crucial during ketogenic diet, and which of their physiological effects are associated with their therapeutic action in migraine, further clinical studies combining biochemical and metabolic assessments are needed. 

Our trial has several limitations. Firstly, the use of a cross over design; this choice was due to the more limited number of subjects required by this type of study design and the greater accuracy due to the comparison of each subject in both conditions. According to the dietary protocol that we adopted (very limited in duration), and to the expected irrelevant diet-related carry-over effect, we decided that each patient should undergo both kinds of diet. However, by the analysis of the average profiles, it is evident that headache clinical features did not return to baseline conditions after the VLCDs, maybe because of the weight reduction, changes to body composition, and a myriad of physiological changes that may have an effect on migraine susceptibility. Nonetheless, the beneficial effect of VLCKD emerged in both groups of patients randomly assigned to each sequence; thus, the study design just was a complication for the interpretations of results, but did not limit the opportunity to detect the efficacy of the VLCKD in comparison to the VLCnKD. Secondly, the number of patients and the duration of treatment periods were limited. The number of patients corresponded, however, to that of the sample size calculation, and was appropriate to show statistical differences in primary and secondary end points. The limited period of dietary intervention was chosen because the company that provided the meal replacements was not willing to provide them for longer periods in a standardized protocol. Even if a one-month duration could be regarded as a too little to observe patients’ full “keto-adaptation” in terms of ketonemia [39] and clinical response, if compared to other forms of headache [40], the short intervention period can nonetheless be regarded as an insignificant limitation, because all headache features improved shortly after the beginning of the diet. Other studies must be performed to investigate the clinical effects of other types of high-fats KDs specifically designed for longer duration, on migraine patients. Third, the very low-calorie diet used is, in principle, not allowed for normo-weighted people. As in our previous study [15], patients were recruited from a dietary clinical setting and not from a headache clinic. This was done because the nutritional protocol we used in the study is a difficult but very effective weight-loss strategy and needs supervision by a dietician. For these reasons, our study is limited to overweight/obese patients. Admittedly, the next step in our exploration of the therapeutic potentialities of ketogenic diet in headaches will be to study its effect in normo-weight episodic and chronic migraine patients. Another limitation of the study is our inability to determine the actual presence of ketonemia in patients. Although we decided to not measure ketonemia and to avoid daily detection, in order to not be influenced and to maintain the blind nature of the study, at the end of the study, we performed ketonuria detection tests by laboratory analyses, which showed that only the 75.9% of patients were positive. This result cannot be interpreted as a lack of validity of the diet to induce a state of ketosis. In fact, urinary analysis does not reflect the ketonemia concentration; rather, it measures the concentration of wasted acetoacetate which, after the glomerular filtration, it is partially reabsorbed by the kidneys, minimizing the body protein loss and maintaining a high circulating ketone concentration [41]. Moreover, in order to standardize the results, all the samples for analysis were collected in the morning on an empty stomach, while it is known that the peak of urinary ketones is in the afternoon [42]. Both conditions made the results of ketonuria detection in our patients an underestimation of the real state of ketosis. In order to better understand diet adherence, in future, blood ketone meters that store a month’s worth of data with the display disabled or covered could be used to provide such data without compromising on blinding.

Finally, we do not regard the knowledge of each label color for patients and investigators as a limitation, since patients were not influenced by the label because they were not in contact with other enrolled patients. They followed the diet and filled the calendars. Investigators were not influenceable by the labeling, since their role was only to collect the calendars, perform the measures, and transmit the data for the database creation and statistical analysis.

## 5. Conclusions

Overall, our results confirm, in a double-blind design, that VLCKD is effective for rapid, short-term improvement of migraines in overweight patients, while VLCnKD is not. Whether this dietary strategy should be applied to all overweight migraine patients and for how long remains to be determined in future studies.

## Figures and Tables

**Table 1:** Micronutrients daily supplemented through integrators by patients.

Micronutrient	Daily Dose	Micronutrient	Daily Dose
Vitamin A	80 mg	Vitamin E	12 mg
Vitamin B1	1.1 mg	Vitamin H	50 mcg
Vitamin B2	1.4 mg	Ca^2+^	800 mg
Vitamin B3	16 mg	Cr^2+^	40 mcg
Vitamin B5	6 mg	Cu^+^	1 mg
Vitamin B6	2 mg	I^-^	150 mcg
Vitamin B8	150 mcg	K^+^	2000 mg
Vitamin B9	200 mcg	Mg^2+^	375 mg
Vitamin B12	2,5 mcg	Mn^2+^	2 mg
Vitamin C	60 mg	Se^4+^	55 mcg
Vitamin D	5 mcg	Zn^2+^	10 mg

**Table 2:** Descriptive and univariate statistics of “R→B” and “B→R” groups at baseline.

Variables	R→B	B→R	Statistics
Gender (women/men)	15/3	14/3	$X_{1}^{2}$ = 0.006, *p* = 1.000
Age (years)	41 (12)	46 (7)	*t*_33_ = −1.661, *p* = 0.106
Headache days/months	6 (3)	8 (4)	*t*_33_ = −1.227, *p* = 0.229
Attacks/months	4.83 (2.01)	5.94 (2.49)	*t*_33_ = −1.454, *p* = 0.155
Symptomatic /months	3.94 (3.59)	5.35 (3.94)	*t*_33_ = −1.107, *p* = 0.276
BMI (Kg/m^2^)	34.9 (5.4)	35.9 (7.2)	*t*_33_ = −0.491, *p* = 0.627

Data are reported as frequencies and means (with SD). BMI, Body Mass Index; “B ≥ R” is the group that followed during the “period 1” the VLCnKD and during the “period 2” the VLCKD; “R ≥ B” is the group that followed during the “period 1” the VLCKD and during the “period 2” the VLCnKD (see Figure 1 for diet sequence). VLCKD: ketogenic very low-calorie diet; VLCnKD: non-ketogenic very low-calorie diet.

**Table 3:** Results of linear mixed-effect models.

Clinical Variables		Estimate	Standard Error	95% CI	df	*t*	*P*
Headache days/months change							
	Treatment	−3.733	0.810	−5.315, −2.148	27	−4.607	0.0001
	Period	0.150	0.810	−1.433, 1.732	27	0.185	0.855
	Gender	0.382	1.313	−2.088, 2.852	25	0.291	0.773
	Age	−0.028	0.042	−0.108, 0.051	25	−0.676	0.505
	BMI	−0.0004	0.073	−0.138, 0.136	25	−0.005	0.996
Attacks/months change							
	Treatment	−3.017	0.581	−4.153, −1.880	27	−5.191	<0.00001
	Period	0.101	0.581	−1.035, 1.236	27	0.173	0.864
	Gender	0.987	0.935	−0.656, 2.630	25	1.056	0.301
	Age	−0.011	0.030	−0.064, 0.042	25	−0.370	0.714
	BMI	−0.030	0.052	−0.121, 0.061	25	−0.576	0.570
Symptomatic drugs/months change							
	Treatment	−1.485	0.903	−3.189, 0.219	27	−1.645	0.112
	Period	0.985	0.903	−0.719, 2.689	27	1.091	0.285
	Gender	0.271	1.306	−2.193, 2.736	25	0.208	0.837
	Age	0.006	0.042	−0.073, 0.085	25	0.145	0.886
	BMI	0.004	0.073	−0.133, 0.141	25	0.051	0.960
BMI change							
	Treatment	−0.204	0.216	−0.606, 0.219	27	−0.943	0.354
	**Period**	1.117	0.216	0.726, 1.511	27	5.162	<0.00001
	Gender	−0.487	0.394	−0.996, 0.081	25	−1.237	0.228
	Age	0.019	0.013	0.002, 0.037	25	1.466	0.155
	BMI	−0.040	0.022	−0.069, −0.009	25	−1.844	0.077

“Treatment” indicates the comparison “VLCKD vs. VLCnKD”, “Period” the comparison “Period 1 vs. Period 2”, “Gender” the comparison “women vs. men”. Overweight patients with migraine had during the VLCKD treatment −3.73 migraine days and −3.02 migraine attacks respect to those patients during VLCnKD treatment. VLCKD: ketogenic very low-calorie diet; VLCnKD: non-ketogenic very low-calorie diet.

## Supplementary Materials

The following are available online at https://www.mdpi.com/2072-6643/11/8/1742/s1, Table S1: Results of blood analysis at T0 (baseline), T1 (the end of the first month of very low-calorie diet) and T3 (the end of the second month of very low calorie diet). Figure S1: Profiles of averages. Means and standard errors of means from T1 to T5 of clinical variables in “R ≥ B” and “B ≥ R” groups. The two VLCD periods were between T1 and T2 and between T3 and T4. Red line indicates VLCKD; blue line indicates VLCnKD.

## Abbreviations

AcAc	Acetoacetate
BHB	Beta-Hydroxybutyrate
BMI	Body Mass Index
CSD	Cortical Spreading Depression
DRI	Dietary Reference Intake
EFSA	European Food Safety Authority
ITT	Intention-to-Treat
KD	Ketogenic Diet
LMM	Linear Mixed Model
PSMF	Protein Sparing Modified Fast
VLCD	Very Low-Calorie Diet
VLCKD	Very Low-Calorie Ketogenic Diet
VLCnKD	Very Low-Calorie non-Ketogenic Diet
